# Renal and Extra Renal Manifestations in Adult Zebrafish Model of Cystinosis

**DOI:** 10.3390/ijms22179398

**Published:** 2021-08-30

**Authors:** Sante Princiero Berlingerio, Junling He, Lies De Groef, Harold Taeter, Tomas Norton, Pieter Baatsen, Sara Cairoli, Bianca Goffredo, Peter de Witte, Lambertus van den Heuvel, Hans J. Baelde, Elena Levtchenko

**Affiliations:** 1 Laboratory of Pediatric Nephrology, KU Leuven, 3000 Leuven, Belgium; santeprinciero.berlingerio@kuleuven.be (S.P.B.); Bert.vandenHeuvel@radboudumc.nl (L.v.d.H.); 2 Department of Pathology, Leiden University Medical Center, 2300 RC Leiden, The Netherlands; J.He@lumc.nl (J.H.); j.j.baelde@lumc.nl (H.J.B.); 3 Neural Circuit Development and Regeneration Research Group, KU Leuven, 3000 Leuven, Belgium; lies.degroef@kuleuven.be; 4 Group of M3-BIORES, Division of Animal and Human Health Engineering, KU Leuven, 3000 Leuven, Belgium; harold.taeter@kuleuven.be (H.T.); tomas.norton@kuleuven.be (T.N.); 5 Molecular Neurobiology, VIB-KU Leuven, 3000 Leuven, Belgium; pieter.baatsen@kuleuven.be; 6 Department of Pediatric Medicine, Laboratory of Metabolic Biochemistry Unit, Bambino Gesù Children’s Hospital, IRCCS, 00146 Rome, Italy; sara.cairoli@opbg.net (S.C.); biancamaria.goffredo@opbg.net (B.G.); 7 Laboratory for Molecular Biodiscovery, KU Leuven, 3000 Leuven, Belgium; peter.dewitte@kuleuven.be; 8 Department of Pediatric Nephrology, Radboud University Medical Center, 6525 GA Nijmegen, The Netherlands

**Keywords:** kidney disease, cystinosis, zebrafish model, renal and extra renal manifestation, adult phenotypic features

## Abstract

Cystinosis is a rare, incurable, autosomal recessive disease caused by mutations in the *CTNS* gene. This gene encodes the lysosomal cystine transporter cystinosin, leading to lysosomal cystine accumulation in all cells of the body, with kidneys being the first affected organs. The current treatment with cysteamine decreases cystine accumulation, but does not reverse the proximal tubular dysfunction, glomerular injury or loss of renal function. In our previous study, we have developed a zebrafish model of cystinosis through a nonsense mutation in the *CTNS* gene and have shown that zebrafish larvae recapitulate the kidney phenotype described in humans. In the current study, we characterized the adult cystinosis zebrafish model and evaluated the long-term effects of the disease on kidney and extra renal organs through biochemical, histological, fertility and locomotor activity studies. We found that the adult cystinosis zebrafish presents cystine accumulation in various organs, altered kidney morphology, impaired skin pigmentation, decreased fertility, altered locomotor activity and ocular anomalies. Overall, our data indicate that the adult cystinosis zebrafish model reproduces several human phenotypes of cystinosis and may be useful for studying pathophysiology and long-term effects of novel therapies.

## 1. Introduction

Cystinosis is an autosomal recessive storage disorder caused by mutations in the *CTNS* gene which encodes the lysosomal cystine proton co-transporter cystinosin, carrying cystine from the lysosomal lumen to the cytosol. Several organs are involved in the disease, such as muscles, testis, eyes and brain, and among all, kidneys are the first and most severely affected organs [1]. The most frequent and severe clinical variant is the infantile nephropathic cystinosis, in which the first clinical manifestation is the renal Fanconi syndrome, characterized by an impaired proximal tubular epithelial cell (PTEC) reabsorption resulting in the urinary loss of amino acids, glucose, low molecular to intermediate molecular weight proteins and other metabolites. The PTEC damage is followed by progressive glomerular dysfunction and, when left untreated, leads to end-stage kidney disease [1]. The current therapeutic approach consists of cysteamine, which is a cystine-depleting amino thiol that breaks the disulfide bridge of cystine, resulting in the formation of amino acid cysteine and a cysteamine-cysteine mixed disulfide. This latter molecule can exit lysosomes via the amino acid transporter PQLC2 [2]. However, treatment with cysteamine, although decreasing lysosomal cystine accumulation, only delays the progression of the disease [3,4,5]. Therefore, further understanding of the pathogenesis of cystinosis and developing new therapeutic options are still on the research agenda. Several animal models have been developed in order to recapitulate the cystinosis phenotype [6,7]. One of the most used models is the Ctns^−/−^ C57BL/6 mouse, which develops cystine accumulation and mild proximal tubulopathy. However, it does not show glomerular damage and the phenotype has been found dependent on the genetic background [8].

To date, zebrafish models have become an attractive tool for investigating human diseases due to several advantages such as their rapid development, high fecundity, lower maintenance cost and the availability of easily applicable gene-editing technologies [9]. In a previous study, we presented a congenic zebrafish larvae model with a homozygous nonsense mutation in the exon 8 of the *ctns^−/−^* gene, resulting in a functional loss of cystinosin [10], leading to cystine accumulation in the entire body, increased larvae deformity, delayed development and kidney damage. However, long-term disease consequences in adult zebrafish have not been studied so far. In the current study, we aimed to characterize the renal and extra renal manifestations in the adult zebrafish model of cystinosis.

## 2. Results

### 2.1. Cystine Accumulates in Ctns^−/−^ Zebrafish

**Figure 1:**
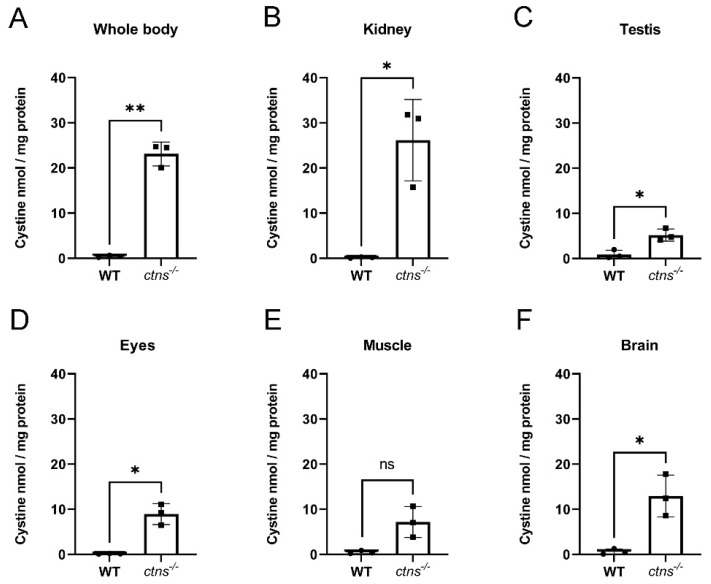
Cystine content in homogenates of wild-type and *ctns^−/−^* zebrafish. (**A**–**F**) Cystine content in whole body (**A**), kidney (**B**), testis (**C**), eyes (**D**), muscle (**E**) and brain (**F**) in wild-type and *ctns^−/−^* 18-month-old male zebrafish. Cystine concentration is expressed as nmol/mg protein. Each dot represents one zebrafish, for a total of *n* = 3 wild-type and *n* = 3 *ctns^−/−^* 18-month-old male zebrafish; unpaired *t* test with Welch’s correction, two tailed: * *p* < 0.05; ** *p* <0.01; ns: not significant.

Cystinosis is considered a multisystemic disorder resulting in cystine accumulation in all cells of the body. In our previous work, we found that cystinosis (*ctns^−/−^*) zebrafish display cystine accumulation starting from the larval stage of development and in several organs up to the age of 8 months [9]. In order to evaluate if our model maintains cystine accumulation at the later stages of the disease, we measured cystine levels in the whole body, kidney, testis, eyes, muscle, and brain in 18-month-old *ctns^−/−^* zebrafish compared with age-matched wild-type male zebrafish. We found a 54-fold increase in cystine content in the whole body (Figure 1A) and a 146-fold increase in the kidney (Figure 1B) of *ctns^−/−^* zebrafish compared with wild-type zebrafish. Moreover, we observed extensive cystine accumulation in testis, eyes, muscle, and brain (Figure 1C–F) in *ctns^−/−^* zebrafish.

### 2.2. Renal Manifestation

#### 2.2.1. *Ctns^−/−^* Zebrafish Present PTEC Damage

**Figure 2:**
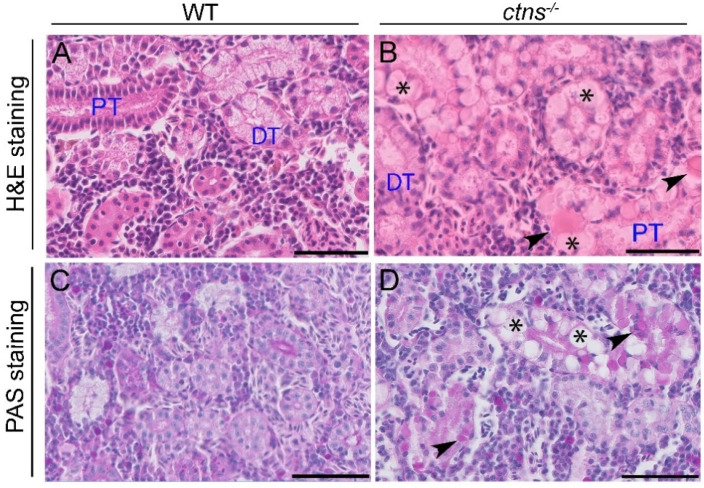
Kidney histology in wild-type and *ctns^−/−^* zebrafish. (**A**,**B**) Representative images of renal tubules of wild-type (**A**) and *ctns^−/−^* (**B**) 18-month-old zebrafish. Details of proximal tubules with hyaline-like eosinophilic droplets (black arrowheads) and cytoplasmic vacuoles (*). H&E staining. The scale bars represent 50 µm. (**C**,**D**) Representative images of the PAS staining of renal tubules of wild-type (**C**) and *ctns^−/−^* (**D**) 18-month-old zebrafish. Details of proximal tubules with hyaline-like droplets (black arrowheads) and cytoplasmic vacuoles (*). PAS staining. The scale bars represent 50 µm. PT: proximal tubule; DT: distal tubule.

**Figure 3:**
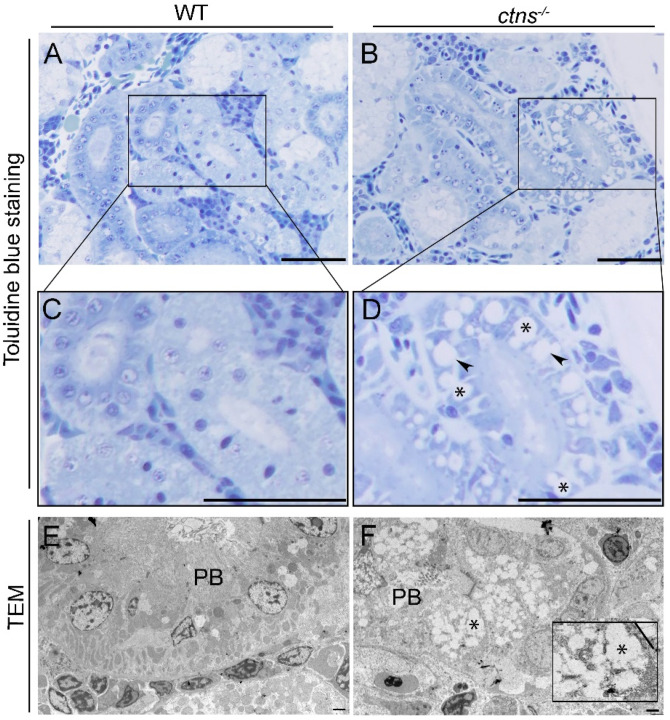
Kidney cystine crystals in wild-type and *ctns^−/−^* zebrafish PTECs. (**A**,**B**) Representative toluidine blue-stained images of renal proximal tubules in wild-type (**A**) and *ctns^−/−^* (**B**) 18-month-old zebrafish. The scale bars represent 50 µm. (**C**,**D**) Figure 3C,D show a higher magnification of Figure 3A,B. Details of renal proximal tubules with cytoplasmic vacuoles ((**D**); *), rectangular and polymorphous vacuolar spaces ((**D**); black arrowheads). (**E**,**F**) Representative TEM images of the renal proximal tubule of wild-type (**E**) and *ctns^−/−^* 18-month-old zebrafish (**F**). (**F**) Partial loss of proximal tubule brush borders (PB) and accumulation of the rectangular and polymorphous vacuoles (*) were observed in the renal proximal tubule of *ctns^−/−^* zebrafish. The high-magnification view of the rectangle in the bottom right corner shows the polymorphous vacuoles and a large vacuole with straight membrane border segments (a straight line). The scale bars represent 5 µm.

Since the kidney is the most severe affected organ, we examined the histological characteristics of the kidney using Hematoxylin and Eosin (H&E) and Periodic Acid-Shiff (PAS) staining. We noted the presence of cloudy swelling, hyaline-like eosinophilic droplets and cytoplasmic vacuoles in the PTEC of *ctns^−/−^* zebrafish (Figure 2B,D) but not in wild-type zebrafish (Figure 2A,C). Of note, no distal tubular damage was found. Cystine crystals were previously observed in PTEC as cytoplasmic vacuoles with rectangular or polymorphous shapes [11]. Therefore, we investigated if these structures were also present in tubules of zebrafish. We found numerous cytoplasmic vacuoles in the PTEC of *ctns^−/−^* zebrafish through toluidine blue staining (Figure 3B,D, indicated with asterisks). In addition, we found vacuolar space with rectangular or polymorphous shapes (Figure 3B,D, indicated with arrowheads). We confirmed this finding by transmission electron microscopy (TEM) (Figure 3F). These structures were not present in the PTEC of wild-type zebrafish (Figure 3A,C,E). Interestingly, in addition to the presence of polymorphous shaped vacuoles, we observed a partial loss of the brush border in PTEC ultrastructure (Figure 3F).

#### 2.2.2. *Ctns^−/−^* Zebrafish Show Glomerular Hypertrophy

**Figure 4:**
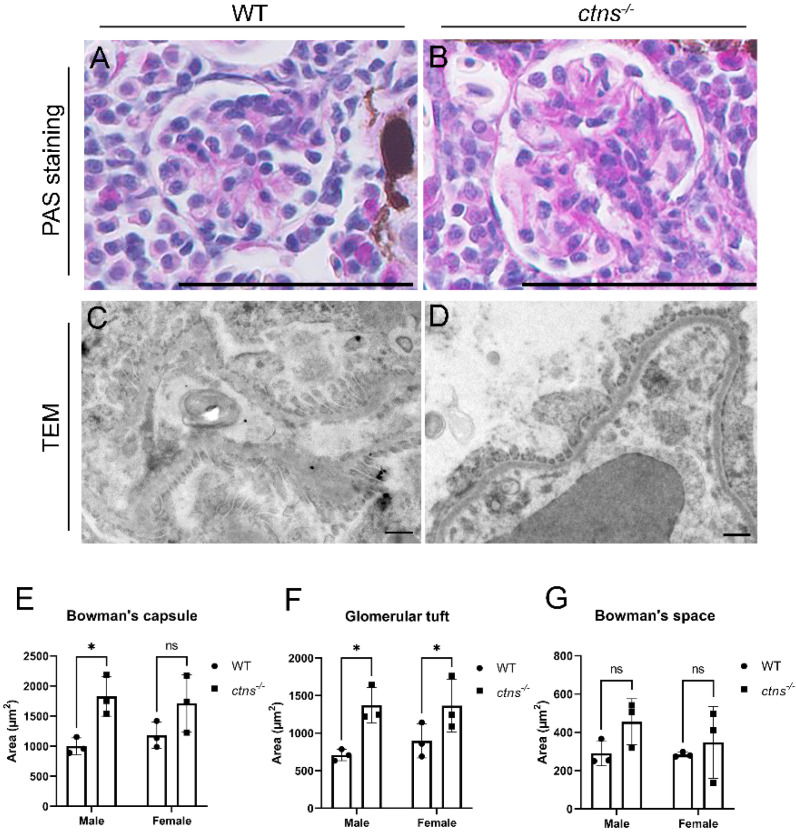
Glomerular morphology in wild-type and in *ctns^−/−^* zebrafish. (**A**,**B**) Representative images of the glomerulus of wild-type (**A**) and *ctns^−/−^* (**B**) 18-month-old zebrafish. PAS staining. The scale bars represent 50 µm. (**C**,**D**) Representative TEM images of the glomerulus of wild-type (**C**) and *ctns^−/−^* (**D**) zebrafish. The scale bars represent 1 µm. (**E**–**G**) The quantification of the surface areas of Bowman’s capsule (**E**), glomerular tuft (**F**), and Bowman’s space (**G**) of wild-type and *ctns^−/−^* zebrafish in both genders. Each dot represents one zebrafish, for a total of *n* = 3 wild-type and *n* = 3 *ctns^−/−^* 18-month-old zebrafish. Two-way ANOVA with Fisher’s least significant difference (PLSD) test: * *p* < 0.05; ns: not significant.

Since glomerular damage usually follows PTEC lesions, we evaluated the glomerular histology of the *ctns^−/−^* zebrafish, and we observed that *ctns^−/−^* male zebrafish showed significant enlargement of Bowman’s capsule (Figure 4B,E) and glomerular tuft (Figure 4B,F), but not Bowman’s space (Figure 4B,G) when compared with wild-type male zebrafish (Figure 4A,E–G). The same was confirmed for the glomerular tuft in female zebrafish carrying the *ctns^−/−^* mutation (Figure 4F), and similar trend was observed upon measurements of the Bowman’s space and the Bowman’s capsule (Figure 4E,G). No proliferation of mesangial cells or glomerulosclerosis were observed. On the TEM images, we did not observe alterations on the glomerular basement membrane, podocyte foot process effacement, or abnormally fenestrated endothelial cells in the glomeruli of *ctns^−/−^* zebrafish (Figure 4D) compared with wild-type zebrafish (Figure 4C).

#### 2.2.3. *Ctns^−/−^* Zebrafish Display Increased Apoptosis at The PTEC Level

**Figure 5:**
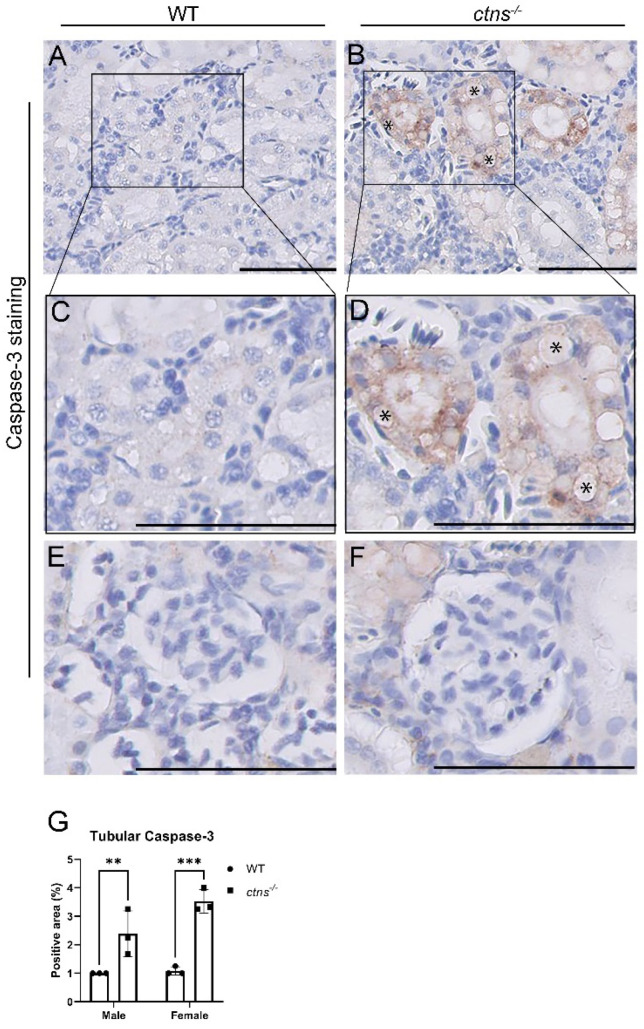
Increased apoptosis in PTECs with vacuoles in *ctns^−/−^* zebrafish. (**A**,**B**) Representative images of the cleaved caspase-3 immunostaining of tubules of wild-type (**A**) and *ctns^−/−^* (**B**) 18-month-old zebrafish. The scale bars represent 50 µm. (**C**,**D**) The bottom panels show a higher magnification of the boxed areas in the upper panels. Details of PTECs with cytoplasmic vacuoles (*). The scale bars represent 50 µm. (**E**,**F**) Representative images of the cleaved caspase-3 immunostaining of glomerulus of wild-type (**E**) and *ctns^−/−^* (**F**) 18-month-old zebrafish. The scale bars represent 50 µm. (**G**) The quantification of the tubular caspase-3 staining positive area in wild-type and *ctns^−/−^* zebrafish in both genders. Each dot represents one zebrafish, for a total of *n* = 3 wild-type and *n* = 3 *ctns^−/−^* 18-month-old zebrafish. Two-way ANOVA with Fisher’s least significant difference (PLSD) test: ** *p* < 0.01; *** *p* < 0.001.

Next, we investigated whether cystinosis triggered cell death in our zebrafish model [10,12]. We performed cleaved caspase-3 immunostaining in the renal tissues of wild-type and *ctns^−/−^* zebrafish and we observed a significant increase of cleaved caspase-3 protein levels in PTEC of *ctns^−/−^* zebrafish (Figure 5B,D,G) compared with wild-type zebrafish (Figure 5A,C,G) in both genders. The expression of cleaved caspase-3 was mainly present in cells showing the polymorphous cytoplasmic vacuoles (Figure 5B,D). No expression of cleaved caspase-3 was present in glomeruli, both in wild-type and *ctns^−/−^* zebrafish (Figure 5E,F).

### 2.3. Extra Renal Manifestations

#### 2.3.1. *Ctns^−/−^* Zebrafish Show a Characteristic Hypopigmented Spotted Skin Pattern

**Figure 6:**
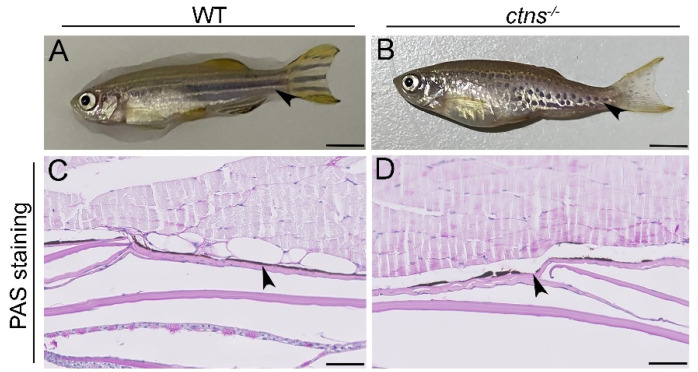
Hypopigmented spotted skin pattern in *ctns^−/−^* zebrafish. (**A**,**B**) Representative pictures of wild-type (**A**) and *ctns^−/−^* (**B**) 18-month-old zebrafish. The scale bars represent 0.5cm. The black arrowheads indicate the different striped skin patterns. (**C**,**D**) PAS-stained pictures of epidermis of wild-type (**C**) and *ctns^−/−^* (**D**) 18-month-old zebrafish. The black arrowheads highlight the melanin layer. The interruption in the melanin layer is present in *ctns^−/−^* (**D**) while it is absent in wild-type zebrafish (**C**). The scale bars represent 50 µm.

Cystinosis patients frequently show premature skin ageing, hypopigmentation, and blond hair [1,13]. Interestingly, we observed a hypopigmented spotted pattern of the skin of *ctns^−/−^* zebrafish, while wild-type fish presented the typical black striped pattern. The phenotype is present in both genders during the entire life (Figure 6A,B). In order to investigate the difference in pigmentation, we performed PAS staining on the zebrafish skin and we found that *ctns^−/−^* zebrafish display altered distribution of melanin layer in the epidermis, which caused the light spotted pattern (Figure 6C,D).

#### 2.3.2. *Ctns^−/−^* Zebrafish Show Spermatogenic Cysts Enriched in Spermatozoa

**Figure 7:**
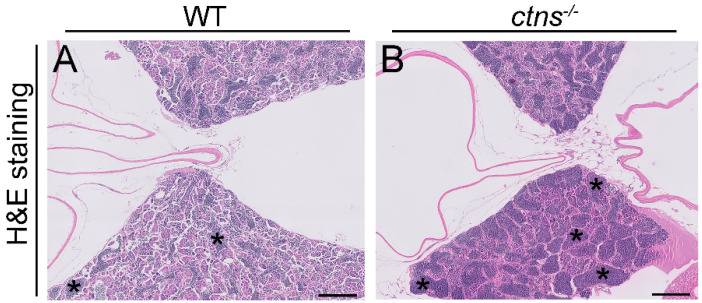
Spermatogenic cysts enriched in spermatozoa in *ctns^−/−^*zebrafish. (**A**,**B**) Representative H&E stained pictures of testis of wild-type (**A**) and *ctns^−/−^*(**B**) 18-month-old zebrafish. Dark blue and rounded staining represent the spermatozoa accumulated in the spermatogenic cysts (indicated with the asterisks). The scale bars represent 200 µm.

Cystinosis is known to affect testicular functions, resulting in azoospermia and infertility in male patients [14,15,16]. The origin of the azoospermia is still unclear, although spermatogenesis has shown to be intact at the testicular level. In contrast with male patients, females with cystinosis are fertile [17]. In our zebrafish model, both female and male *ctns^−/−^*zebrafish are fertile. In order to investigate whether *ctns^−/−^*mutation affects the anatomy of the testis, we performed PAS staining and we found increased accumulation of spermatozoa in the spermatogenic cysts in *ctns^−/−^*zebrafish males (Figure 7B) when compared with wild-type zebrafish males (Figure 7A).

#### 2.3.3. *Ctns^−/−^* Female Zebrafish Show Decreased Egg Production

**Figure 8:**
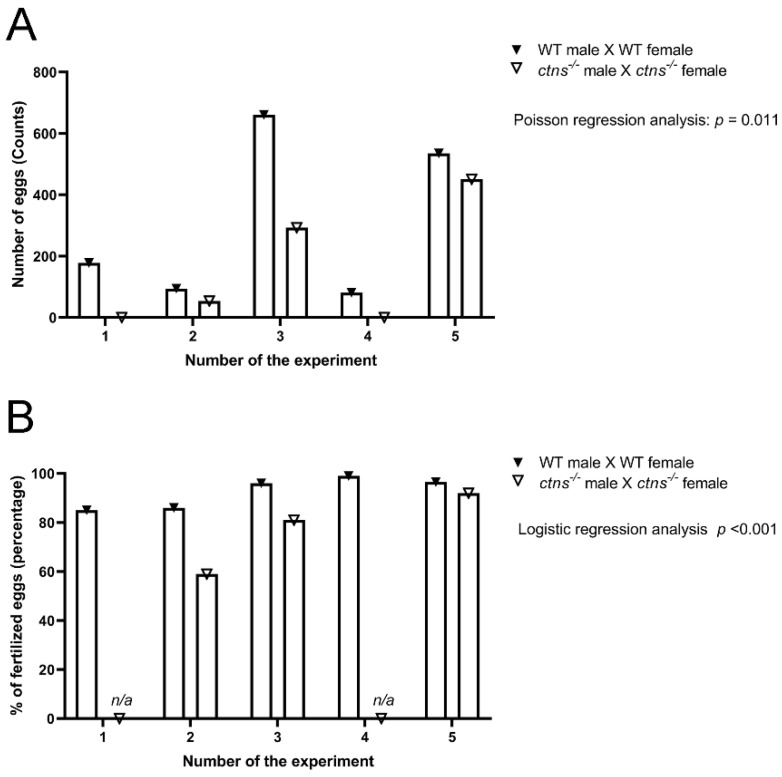
Egg production and percentage of fertilized eggs in wild-type and *ctns^−/−^*zebrafish. (**A**) Egg production was measured as a total number of eggs in wild type and *ctns^−/−^*zebrafish 18-month-old zebrafish. The egg production rate is 1.941 high in wild-type zebrafish compared with *ctns^−/−^*18-month-old zebrafish (rate ratio = 1.941); 95% CI (lower bound = 1.165, higher bound = 3.234); Poisson regression analysis; *p* = 0.011. (**B**) The percentage of fertilized eggs was measured on the total number of eggs. The odds of the fertilized eggs are 3.504 high in wild-type zebrafish compared with *ctns^−/−^*18-months-old zebrafish (odds ratio = 3.504); 95% CI (lower bound = 2.005, higher bound = 6.124); Logistic regression analysis; *p* < 0.001; *n/a*: not applicable.

In order to investigate the functionality of the reproductive system, we measured egg production and the percentage of fertilized eggs in both wild-type and *ctns^−/−^* zebrafish. We found that *ctns^−/−^*females show a decreased egg production (Figure 8A) and decreased percentage of fertilized eggs (Figure 8B) compared with the wild-type females.

#### 2.3.4. *Ctns^−/−^* Zebrafish Display Decreased Locomotor Activity

**Figure 9:**
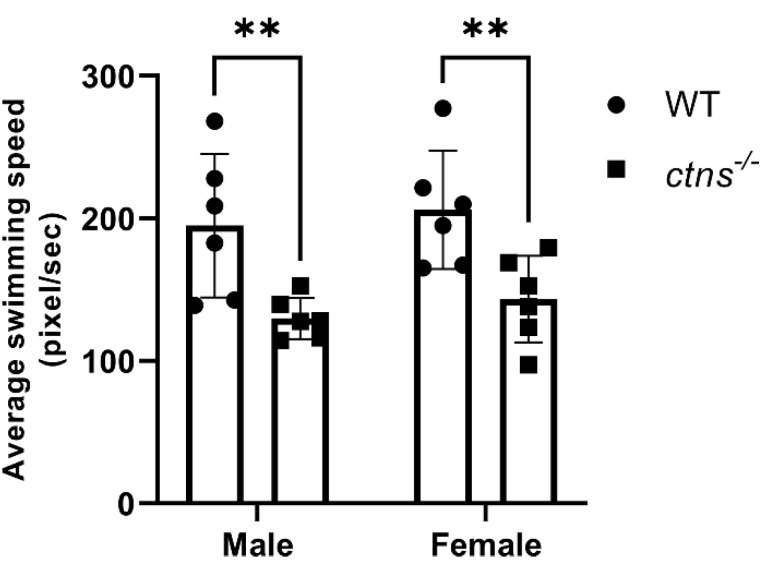
Average swimming speed in wild-type and *ctns^−/−^*zebrafish. The average speed is decreased in *ctns^−/−^*zebrafish compared with wild-type in both genders. The average swimming speed is measured in pixel/second. Each dot represents one zebrafish, for a total of *n* = 6 wild-type and *n* = 6 *ctns^−/−^*18-month-old zebrafish. Two-way ANOVA with Fisher’s least significant difference (PLSD) test: ** *p* < 0.01.

Since muscle wasting and weakness have been diagnosed in cystinosis patients, generally from the second decade of life [1,18], we evaluated the locomotor activity in our zebrafish model. We discovered that *ctns^−/−^*zebrafish from both genders showed decreased locomotor activity compared with wild-type zebrafish, suggesting an impaired muscle function (Figure 9). However, histological analysis of skeletal muscle tissue did not reveal apparent abnormalities in *ctns^−/−^*zebrafish.

#### 2.3.5. *Ctns^−/−^* Zebrafish Present Increased Thickness of THE Cornea

**Figure 10:**
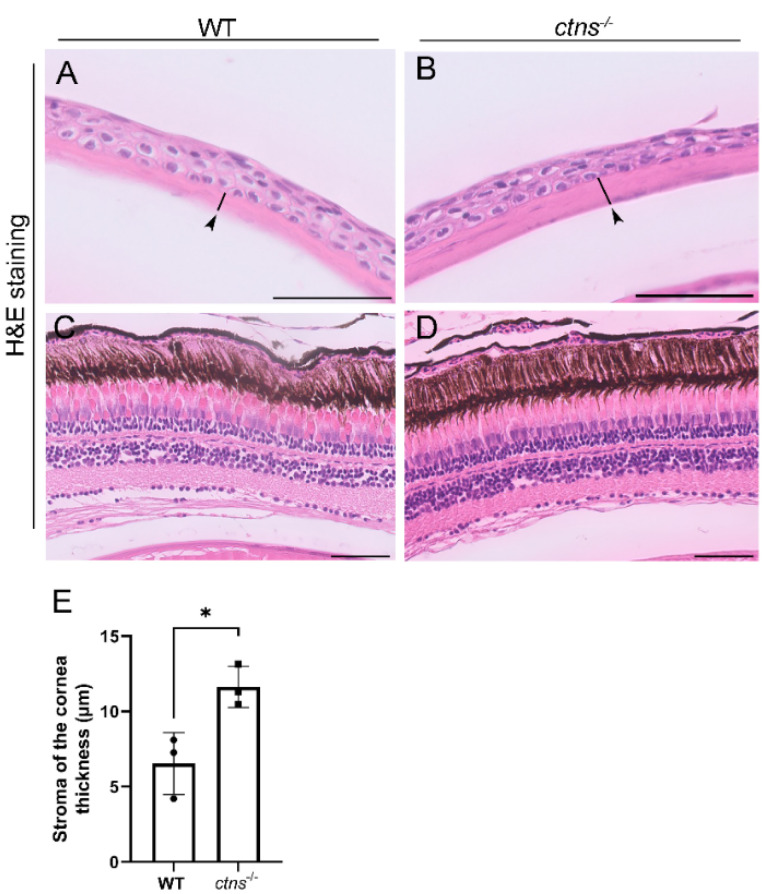
Eye histology in wild-type and *ctns^−/−^*zebrafish. (**A**,**B**) Representative images of cornea of wild-type (**A**) and *ctns^−/−^*(**B**) 18-month-old zebrafish. The stromal layer of the cornea (black arrowheads and black lines). H&E staining. The scale bars represent 50 µm. (**C**,**D**) Representative images of retina of wild-type (**A**) and *ctns^−/−^*(**B**) zebrafish. H&E staining. The scale bars represent 50 µm. (**E**) Relative quantification of the thickness of the stromal layer of the cornea. Each dot represents one zebrafish, for a total of *n* = 3 wild-type and *n* = 3 *ctns^−/−^*18-month-old zebrafish. Unpaired *t* test with Welch’s correction, two tailed: * *p* < 0.05.

Cystinosis affects eyes resulting in cystine accumulation in several tissues, such as iris, conjunctiva and retinal epithelium, however, cornea is the most affected part due to cystine accumulation [19] and presents increased thickness [20]. Using H&E staining, we noted that *ctns^−/−^*zebrafish showed increased thickness of the stromal layer of the cornea compared with wild-type (Figure 10A,B,E) zebrafish. However, we did not reveal apparent abnormalities in the retinal epithelium of *ctns^−/−^*zebrafish (Figure 10C,D).

#### 2.3.6. Additional Ctns^−/−^ Zebrafish Phenotypes

In addition to the phenotypes mentioned above, we performed histological analysis of the brain and ovary without finding differences between *ctns^−/−^*and wild-type zebrafish. Moreover, we evaluated body length and weight of *ctns^−/−^*and wild-type zebrafish at 18 months old and we found an increased body weight in *ctns^−/−^*zebrafish while we found an increased body length in *ctns^−/−^*zebrafish of both genders compared with wild-type (Supplementary Figure S1).

## 3. Discussion

During the last decades, cystinosis has transformed from a fatal disease of childhood into a treatable metabolic disorder with which patients can survive into adulthood and reach advanced age [21,22]. Longer survival of the patients revealed novel disease phenotypes and raised new questions regarding the pathogenesis and long-term effects of therapies. In this regard, studying adult animal models of cystinosis becomes more and more relevant.

In the current study, we investigated the renal and extra renal manifestations of cystinosis in an adult zebrafish model of 18 months old, which corresponds to the human age of 40–50 years [23,24]. First, we validated that the adult *ctns^−/−^* zebrafish model presents the whole-body and various organs cystine accumulation. In the kidney, at the histological level, we observed signs of PTEC damage reflected by the presence of numerous hyaline-like eosinophilic droplets, cytoplasmic vacuoles and partial loss of brush borders. Furthermore, the *ctns^−/−^*male zebrafish developed glomerular hypertrophy. Lastly, we showed that cleaved-caspase 3 expression was increased in the renal PTEC of *ctns^−/−^*zebrafish, indicating that apoptosis is involved in the pathogenesis of nephropathic cystinosis in zebrafish. Since cystinosis patients present also extra renal manifestations, we investigated the effect of *ctns^−/−^* mutation in other organs in our zebrafish model. Interestingly, we found that *ctns^−/−^*mutation caused an impairment in the skin anatomy and affected fertility, locomotor activity and eyes.

Lysosomes are the sites of intracellular digestion and are considered to be the vital coordinators of cell metabolism [25]. Being a lysosomal storage disease, the key feature of cystinosis is lysosomal cystine accumulation [26,27]. Interestingly, we found a 54-fold increase of cystine in the whole-body of the 18-months cystinosis zebrafish when compared with wild-type, while, in our previous study [10], cystinosis zebrafish larvae at 6 days post fertilization presented a 13-fold increase. Overall, these data suggest that cystine accumulates during the lifetime in cystinosis zebrafish. Liquid chromatography and mass spectrometry analysis have confirmed that, among all organs, kidneys are the preferred site for cystine accumulation. Lysosomal swelling and hyaline-like eosinophilic droplets inside the lysosomes have been reported in cystine-loaded PTEC [28,29] and, in human biopsies, cystine crystals appear as polymorphous shape spaces within the interstitial macrophages and the PTEC cytoplasm [27]. In human tissue, cystine accumulation and crystallization is a cumulative process occurring during the lifetime. In line, cystine crystals were not detected in the *ctns^−/−^*zebrafish at the larval stage [10] while, in our pilot experiments, we found that the polymorphous shape of cystine crystals appear at the age of 3 and 6 months and are clearly visible at the age of 18-months, suggesting a progressive worsening of the effects of cystinosis in the kidney.

To assess the histological changes of the glomeruli, we measured the surface areas of Bowman’s capsule, the glomerular tuft and Bowman’s space of *ctns^−/−^*zebrafish on PAS-stained sections. We found that *ctns^−/−^*zebrafish developed glomerular hypertrophy, suggesting a hyperfiltration. The mild glomerular damage observed in the *ctns^−/−^*zebrafish might be attributed to the regenerative ability of zebrafish kidney, being able to add new nephrons, as well as repairing existing nephrons [30].

In addition to the histological damage, cystine accumulation has been shown to activate protein kinase C triggering apoptosis in PTEC [12]. Thus, we evaluated the cleaved caspase-3 expression in PTEC and we found that the *ctns^−/−^*zebrafish presented increased caspase-3 and nuclear fragmentation. The presence of cleaved caspase-3 was mainly found in cells that accumulated the cytoplasmic vacuoles, suggesting that apoptosis takes place in the injured PTEC. One additional mechanism that can explain the increased apoptosis might be the presence of reactive oxygen species due to reduced glutathione associated with cystine accumulation [31]. However, further studies are necessary to evaluate if this mechanism occurs also in *ctns^−/−^*zebrafish.

Although kidneys are the first and most severely affected organs, cystinosis is a multi-systemic disease in which various organs, such as skin, gonads, eyes and muscles are involved. From literature, it is known that cystinosis patients frequently display premature skin ageing, blond hair and fair skin color [13]. The latter phenotype is due to the involvement of cystinosin transporter in the regulation of melanin synthesis [32] and in the maintenance of darker pigmentation through the newly discovered cysteine transporter MFSD12 [33]. Excitingly, our adult *ctns^−/−^*zebrafish model presented a hypopigmented and spotted skin pattern and showed an impaired melanin distribution which was not observed at the larval stage [10].

In addition, cystinosis causes infertility in males, while females are known to be fertile [14]. Recent data suggest that infertility could be due to progressive testicular degeneration, leading to altered semen quality or due to an obstruction in an undefined part of the male excretory system. The obstructed structures might affect the quality of the semen, especially in the regions where ducts have a very small diameter, for instance, the rete testis [16]. In zebrafish, the spermatogenic process is very similar to mammals; however, one of the main differences is that spermatogenesis occurs in the cysts [34]. In our study, we found increased spermatozoa in the spermatogenic cyst of *ctns^−/−^*zebrafish, however, we did not find signs of testicular degeneration. Therefore, the described phenotype could suggest an obstruction at testicular levels that leads to less release of spermatozoa in the environment, however further studies are necessary to confirm this hypothesis. At the functional level, the decreased egg numbers and decreased fertilized eggs seem to reflect an impairment in the fertility of *ctns^−/−^*zebrafish.

Another clinical manifestation of cystinosis, generally affecting patients starting from the second decade of life, is myopathy, characterized by muscle wasting and weakness [1]. Interestingly, we found that *ctns^−/−^*adult zebrafish present decreased locomotor activity compared with control, suggesting an impaired muscular function. Of note, this phenotype was not present in larvae [10], suggesting that it affects *ctns^−/−^*zebrafish during the later stage of the disease. Despite this, no visible histological differences were detected in the muscles. The decreased locomotor activity might be due to mitochondrial dysfunction which could not be detected by routine histology studies [35,36,37]. Hence, a comprehensive characterization of mitochondria in cystinosis zebrafish needs further evaluation.

In addition to the above-mentioned phenotypes, several studies have shown that cystinosis patients also display ocular symptoms such as photophobia and blepharospasm [1], with cornea being one of the most affected parts of the eye resulting in cystine crystal accumulation and increased thickness [20]. However, little is known about the causes of the increased thickness of the cornea. For instance, it was suggested that the described phenotype is due to a subclinical dysfunction of epithelial and/or endothelial cells leading to stromal edema [20]. In our study, we found that *ctns^−/−^*adult zebrafish display increased thickness of the stromal layer of the cornea, indicating that our model shows signs of cornea dysfunction in cystinosis.

One of the main gaps to fill for a comprehensive understanding of cystinosis is the lack of an animal model that fully recapitulates the human disease. Several mouse models have been developed, showing cystine accumulation but failing to reproduce the complete renal phenotype [8,26]. Specifically, the C57BL/6 mouse presented cystine accumulation in several organs, which increases with age [8]. In addition, the C57BL/6 mouse model shows proximal tubular lesions starting from the age of 6 months. Similarly, in our model, proximal tubular lesions worsen over time. However, at the glomerular level, the C57BL/6 mouse model presented a normal phenotype, while our adult zebrafish model shows glomerular hypertrophy. In addition, the C57BL/6 mouse model fails to mimic the impaired fertility observed in humans, while the *ctns^−/−^*zebrafish model presents spermatogenic cysts enriched in spermatozoa and decreased eggs production. At the ocular level, both mouse [38] and the *ctns^−/−^*zebrafish model presents ocular abnormalities. On the contrary, at the skin level, this mouse model does not show impaired melatonin production [8], which appears in the adult zebrafish model as a hypopigmented skin pattern. Lastly, at the behavioral level, our *ctns^−/−^* zebrafish model presents a decreased locomotor activity, which may be caused by an impaired mitochondrial function or it might be caused by a secondary effect of the increased weight in cystinosis zebrafish. Overall, it seems that our *ctns^−/−^* zebrafish model better recapitulates the human disease. Recently, Shimizu et al. established a novel congenic *Ctns^ugl^* mutation in a rat strain that presented renal lesions and cystine crystals in the lysosomes of the kidney cortex [7]. However, a more detailed characterization of the model is still missing. Therefore, there is an urgent need to develop and characterize new functional models that recapitulate human disease.

In conclusion, we demonstrated that our adult *ctns^−/−^* zebrafish model reproduces several human phenotypes of cystinosis and it may be useful for studying the pathogenesis of the disease in adults and for testing long-term effects of novel drugs for correcting renal and extra-renal manifestations.

## 4. Materials and Methods

### 4.1. Zebrafish Mainteinance and Breeding

Zebrafish were handled and maintained in compliance with the KU Leuven animal welfare regulations (Ethical approval nr.142/2019). For this study, we included *ctns^−/−^*zebrafish at 18 months old and the wild-type control zebrafish in both genders. The details of generating the *ctns^−/−^*zebrafish were described in our previous study [10].

### 4.2. Cystine Measurement

Cystine levels were measured in zebrafish (wild-type male fish, *n* = 3 and *ctns^−/−^*male fish, *n* = 3) and the amount was expressed as nmol/mg proteins. First, zebrafish were sacrificed with immersion in tricaine methane sulfonate MS-222 (300 mg/L), afterwards, tissue samples were sonicated in the presence of 200 μL of 5 mM N-ethylmaleimide (NEM, St. Louis, MO, USA, Sigma-Aldrich E3876) in Dulbecco’s phosphate buffered saline (dPBS), for a maximum of 200 mg of tissue for each lysate. Subsequently, 100 μL of 12% sulfosalicylic acid (SSA) were added to each homogenate and samples were centrifuged at 12,000 *g* for 10 min, 4 °C. Supernatants containing cystine were stored at −80 °C until time of analysis, while pellets were dissolved overnight at 4 °C in 300 μL of 0.1 M NaOH and kept at − 80 °C until protein is measured using Pierce BCA Protein Assay Reagent Kit. Subsequently, the amount (nmol) of cystine was normalized to the quantity (mg) of protein from each sample. Next, 50 μL of the supernatant containing cystine was spiked with 50μL of the internal standard solution (Cystine d6) and vortexed for 5 s; then the mixture was extracted with 200 μL of acetonitrile, vortexed for 30 s, and then centrifuged at 16,000 *g* for 9 min. Liquid chromatography and mass spectrometry analysis was performed by a UHPLC Agilent 1290 Infinity II 6470 (Agilent Technologies, Santa Clara, CA, USA)equipped with an ESI-JET-STREAM source operating in the positive ion (ESI+) mode. The software used for controlling this equipment and analyzing data was MassHunter (Work station Agilent Technologies, Barcelona, Spain). The separation column used was InfinityLab Poroshell 120 HILIC 1.9 μm 100 × 2.1 mm(Agilent Technologies, Santa Clara, CA, USA).

### 4.3. Hematoxilin and Eosin and Periodic Acid-Shiff Staining

Zebrafish (wild-type female fish, *n* = 3; wild-type male fish, *n* = 3; *ctns^−/−^*female fish, *n* = 3; *ctns^−/−^*male fish, *n* = 3) were sacrificed with immersion in tricaine methane sulfonate MS-222 (300 mg/L), afterwards, the whole zebrafish were fixed in 4% buffered paraformaldehyde (4% PFA) at 4 °C for 1 week. After being washed with dPBS twice, the zebrafish were transferred to EDTA solution (100 mM, pH = 8) for the decalcification for another 1 week, followed by embedding into paraffin. Paraffin-embedded zebrafish tissue was cut (4-µm thickness) on a Leica microtome(Leica Biosystems, Wetzlar, Germany). Sections were stained with H&E and PAS according to the standard protocols.

For histological studies of zebrafish eyes (wild-type female fish, *n* = 3 and *ctns^−/−^*female fish, *n* = 3), eyes were enucleated and fixed in 4% PFA at room temperature for 4 h. Following embedding into optimal cutting temperare medium(Tissue-Tek^®^ VIP^®^; Sakura Finetek, Japan), 10-µm cryosections were made(Cryostar NX70, Thermofisher Scientific, Tokyo, Japan). Sections were stained with H&E according to the standard protocols. Images were made with a Leica brightfield microscope(Leica DM6, Wetzlar, Germany).

### 4.4. Immunohistochemistry (Cleaved Caspase-3 Staining)

Sections with the fresh-cut zebrafish tissue were deparaffinized and rehydrated. Sections were subjected to heat-induced antigen retrieval using 10 mM citrate buffer (pH = 6). After blocking with 5% Normal Goat Serum (NGS) in dPBS, the sections were incubated with rabbit anti-cleaved caspase-3 (Cell Signaling, Danvers, MA, USA, 1:200 dilution) followed by an anti-rabbit-Envision, HRP-labelled secondary antibody(Dako Products, Agilent, Santa Clara, CA, USA). As a negative control, a normal rabbit serum was used. Diaminobenzidine (DAB, DAKO, Glostrup, Denmark) was used as the chromogen. Subsequently, sections were counterstained with H&E, dehydrated and mounted.

### 4.5. Toluidine Blue Staining and TEM

Zebrafish renal tissues (wild-type male zebrafish, *n* = 3 and *ctns^−/−^*male zebrafish, *n* = 3) were harvested and fixed in the EM fixation buffer (1.5% glutaraldehyde/1% paraformaldehyde) for 24 h. Subsequently, the renal tissues were post-fixed with 2.5% glutaraldehyde/1.2% acrolein in fixation buffer (0.1 mol/L cacodylate, 0.1 mol/L sucrose, pH 7.4) and 1% osmium tetroxide, and embedded into epon resin. Semi-thin sections (0.5-µm thickness) were stained with toluidine blue. The ultrathin sections were stained with uranyl acetate. The images were collected using a JEM-1200 EX transmission electron microscopy (JEOL ltd, Tokyo, Japan) with different magnifications.

### 4.6. Digital Image Analysis

Stained slides were digitized using a Philips Ultra-Fast Scanner 1.6 RA (Philips, Eindhoven, The Netherlands)

#### 4.6.1. Glomerular Hypertrophy Analysis

To analyze glomerular hypertrophy in zebrafish, the surface areas (μm^2^) of Bowman’s capsule, Bowman’s space, and glomerular tuft was measured on PAS-stained slides. All available glomeruli per section were included and measured using ImageJ software. The average of the measurements from each zebrafish was used for statistical analysis.

#### 4.6.2. Cleaved Caspase-3 Expression Analysis

Two observers scored the cleaved caspase-3 expression in the tubules of each zebrafish. The semiquantitative score was conducted on the three randomized tubular fields in each zebrafish (20× magnification). The percentage of caspase-3 positive area relative to the total area of tubules was scored as 1 (negative staining), 2 (1–10% positive staining), 3 (10%–25% positive staining), 4 (>25% positive staining). The mean of the score from each zebrafish was used for statistical analysis.

#### 4.6.3. Thickness of the Corneal Stroma Analysis

The thickness of the corneal stroma of zebrafish (10× magnification) was measured at the same distances from the center of the eye by using ImageJ software on zebrafish. For each picture, *n* = 7 measurements at different locations in the eye section (central, middle, periphery) were taken along the cornea at interval of 100 μM. The average of the measurements was used for statistical analysis.

### 4.7. Fertility Study

The fertility was assessed by mating female and male zebrafish (wild-type male fish, *n* = 4 and wild-type female fish, *n* = 6 and *ctns^−/−^*male fish, *n* = 4 and *ctns^−/−^*female fish, *n* = 6) in a spawning tray. In the next morning, one hour after the light, embryos were collected and the total number of eggs produced was recorded. Fertilized eggs were screened and counted from the unfertile ones using a light microscopy. The breeding was performed *n* = 7 times once a week in a row. Two experiments resulting in no egg productions simultaneously in both wild-type and *ctns^−/−^*zebrafish were excluded.

### 4.8. Locomotory Activity

Zebrafish (wild-type female fish, *n* = 6; wild-type male fish, *n* = 6; *ctns^−/−^*female fish, *n* = 6; *ctns^−/−^*male fish, *n* = 6) were placed in the regular housing units and, after an adaptation time of 5 min, were filmed for 5 min. Video recording was executed using a GoPro^®^ HERO 7 placed on top of the tank, thus offering a top view of the fish swimming. Resolution and frame rate were set respectively to 1080 p (1920 × 1080) and 30 fps. Videos were analyzed with idTracker.ai tracking the trajectories of individual zebrafish throughout the experiment. The trajectories were subsequently analyzed with custom python code to derive the average locomotor speed (pixel/sec) in each group [39].

### 4.9. Body Weight and Length Measurements

Zebrafish were euthanized with immersion in 300 mg/L of tricaine methane sulfonate MS-222. After the verification of the absence of response to external stimuli, the euthanized zebrafish were placed on a paper tissue and dried. The length of the euthanized zebrafish was evaluated using a caliper, measuring from the tip of the mouth to the caudal peduncle. The weight of the euthanized zebrafish was evaluated with an analytical scale.

### 4.10. Statistical Analysis

Statistical analysis was performed using SPSS (IBM, New York, NY, USA) and Graphpad Prism (GraphPad Software, La Jolla). Data of two groups were analyzed using unpaired *t* test with Welch’s correction, two tailed. Data of two categorical independent variables were analyzed with two-way ANOVA with Fisher’s least significant difference (PLSD) test. Differences with *p* < 0.05 were considered as statistically significant.

### 4.11. Fertility Study

For the comparison of the rates of egg production between wild-type and *ctns^−/−^*zebrafish, a Poisson regression analysis using Generalized Estimating Equations with robust standard errors to take account of the correlation of rates within experiments was performed. For the comparison of the odds of fertilized eggs between wild-type and *ctns^−/−^*zebrafish A logistic regression analysis using Generalized Estimating Equations with robust standard errors to account for the correlation of the odds within experiments was performed.

## Supplementary Materials

The following are available online at https://www.mdpi.com/article/10.3390/ijms22179398/s1.

## References

[1] Elmonem M.A., Veys K.R., Soliman N.A., van Dyck M., van den Heuvel L.P., Levtchenko E. (2016). Cystinosis: A review. Orphanet J. Rare Dis..

[2] Jezegou A., Llinares E., Anne C., Kieffer-Jaquinod S., O’Regan S., Aupetit J., Chabli A., Sagne C., Debacker C., Chadefaux-Vekemans B. (2012). Heptahelical protein PQLC2 is a lysosomal cationic amino acid exporter underlying the action of cysteamine in cystinosis therapy. Proc. Natl. Acad. Sci. USA.

[3] Besouw M.T., Levtchenko E.N. (2014). Improving the prognosis of nephropathic cystinosis. Int. J. Nephrol. Renovasc. Dis..

[4] Brodin-Sartorius A., Tete M.J., Niaudet P., Antignac C., Guest G., Ottolenghi C., Charbit M., Moyse D., Legendre C., Lesavre P. (2012). Cysteamine therapy delays the progression of nephropathic cystinosis in late adolescents and adults. Kidney Int..

[5] Cherqui S. (2012). Cysteamine therapy: A treatment for cystinosis, not a cure. Kidney Int..

[6] David D., Princiero Berlingerio S., Elmonem M.A., Oliveira Arcolino F., Soliman N., van den Heuvel B., Gijsbers R., Levtchenko E. (2019). Molecular Basis of Cystinosis: Geographic Distribution, Functional Consequences of Mutations in the CTNS Gene, and Potential for Repair. Nephron.

[7] Shimizu Y., Yanobu-Takanashi R., Nakano K., Hamase K., Shimizu T., Okamura T. (2019). A deletion in the Ctns gene causes renal tubular dysfunction and cystine accumulation in LEA/Tohm rats. Mamm. Genome.

[8] Nevo N., Chol M., Bailleux A., Kalatzis V., Morisset L., Devuyst O., Gubler M.C., Antignac C. (2010). Renal phenotype of the cystinosis mouse model is dependent upon genetic background. Nephrol. Dial. Transplant.

[9] Elmonem M.A., Berlingerio S.P., van den Heuvel L.P., de Witte P.A., Lowe M., Levtchenko E.N. (2018). Genetic Renal Diseases: The Emerging Role of Zebrafish Models. Cells.

[10] Elmonem M.A., Khalil R., Khodaparast L., Khodaparast L., Arcolino F.O., Morgan J., Pastore A., Tylzanowski P., Ny A., Lowe M. (2017). Cystinosis (ctns) zebrafish mutant shows pronephric glomerular and tubular dysfunction. Sci. Rep..

[11] Lusco M.A., Najafian B., Alpers C.E., Fogo A.B. (2017). AJKD Atlas of Renal Pathology: Cystinosis. Am. J. Kidney Dis..

[12] Park M.A., Pejovic V., Kerisit K.G., Junius S., Thoene J.G. (2006). Increased apoptosis in cystinotic fibroblasts and renal proximal tubule epithelial cells results from cysteinylation of protein kinase Cdelta. J. Am. Soc. Nephrol..

[13] Veys K.R.P., Elmonem M.A., Dhaenens F., Van Dyck M., Janssen M., Cornelissen E.A.M., Hohenfellner K., Reda A., Quatresooz P., van den Heuvel B. (2019). Enhanced Intrinsic Skin Aging in Nephropathic Cystinosis Assessed by High-Definition Optical Coherence Tomography. J. Invest. Dermatol..

[14] Besouw M.T., Kremer J.A., Janssen M.C., Levtchenko E.N. (2010). Fertility status in male cystinosis patients treated with cysteamine. Fertil. Steril..

[15] Veys K.R., D’Hauwers K.W., van Dongen A., Janssen M.C., Besouw M.T.P., Goossens E., van den Heuvel L.P., Wetzels A., Levtchenko E.N. (2018). First Successful Conception Induced by a Male Cystinosis Patient. JIMD Rep..

[16] Rohayem J., Haffner D., Cremers J.F., Huss S., Wistuba J., Weitzel D., Kliesch S., Hohenfellner K. (2021). Testicular function in males with infantile nephropathic cystinosis. Hum. Reprod..

[17] Blakey H., Proudfoot-Jones J., Knox E., Lipkin G. (2019). Pregnancy in women with cystinosis. Clin. Kidney J..

[18] Dogulu C.F., Tsilou E., Rubin B., Fitzgibbon E.J., Kaiser-Kupper M.I., Rennert O.M., Gahl W.A. (2004). Idiopathic intracranial hypertension in cystinosis. J. Pediatr..

[19] Dixon P., Christopher K., Chauhan A. (2018). Potential role of stromal collagen in cystine crystallization in cystinosis patients. Int. J. Pharm..

[20] Katz B., Melles R.B., Schneider J.A., Rao N.A. (1989). Corneal thickness in nephropathic cystinosis. Br. J. Ophthalmol..

[21] Nesterova G., Gahl W.A. (2013). Cystinosis: The evolution of a treatable disease. Pediatr. Nephrol..

[22] Ariceta G., Giordano V., Santos F. (2019). Effects of long-term cysteamine treatment in patients with cystinosis. Pediatr. Nephrol..

[23] Gilbert M.J., Zerulla T.C., Tierney K.B. (2014). Zebrafish (Danio rerio) as a model for the study of aging and exercise: Physical ability and trainability decrease with age. Exp. Gerontol..

[24] Gerhard G.S., Kauffman E.J., Wang X., Stewart R., Moore J.L., Kasales C.J., Demidenko E., Cheng K.C. (2002). Life spans and senescent phenotypes in two strains of Zebrafish (Danio rerio). Exp. Gerontol..

[25] Saftig P., Klumperman J. (2009). Lysosome biogenesis and lysosomal membrane proteins: Trafficking meets function. Nat. Rev. Mol. Cell Biol..

[26] Cherqui S., Sevin C., Hamard G., Kalatzis V., Sich M., Pequignot M.O., Gogat K., Abitbol M., Broyer M., Gubler M.C. (2002). Intralysosomal cystine accumulation in mice lacking cystinosin, the protein defective in cystinosis. Mol. Cell. Biol..

[27] Raggi C., Luciani A., Nevo N., Antignac C., Terryn S., Devuyst O. (2014). Dedifferentiation and aberrations of the endolysosomal compartment characterize the early stage of nephropathic cystinosis. Hum. Mol. Genet..

[28] Hard G.C. (2008). Some aids to histological recognition of hyaline droplet nephropathy in ninety-day toxicity studies. Toxicol. Pathol..

[29] Sakarcan A., Timmons C., Baum M. (1994). Intracellular distribution of cystine in cystine-loaded proximal tubules. Pediatr. Res..

[30] Kroeger P.T., Wingert R.A. (2014). Using zebrafish to study podocyte genesis during kidney development and regeneration. Genesis..

[31] Sumayao R., McEvoy B., Newsholme P., McMorrow T. (2016). Lysosomal cystine accumulation promotes mitochondrial depolarization and induction of redox-sensitive genes in human kidney proximal tubular cells. J. Physiol..

[32] Chiaverini C., Sillard L., Flori E., Ito S., Briganti S., Wakamatsu K., Fontas E., Berard E., Cailliez M., Cochat P. (2012). Cystinosin is a melanosomal protein that regulates melanin synthesis. FASEB J..

[33] Adelmann C.H., Traunbauer A.K., Chen B., Condon K.J., Chan S.H., Kunchok T., Lewis C.A., Sabatini D.M. (2020). MFSD12 mediates the import of cysteine into melanosomes and lysosomes. Nature.

[34] Leal M.C., Cardoso E.R., Nobrega R.H., Batlouni S.R., Bogerd J., Franca L.R., Schulz R.W. (2009). Histological and stereological evaluation of zebrafish (Danio rerio) spermatogenesis with an emphasis on spermatogonial generations. Biol. Reprod..

[35] Sansanwal P., Sarwal M.M. (2010). Abnormal mitochondrial autophagy in nephropathic cystinosis. Autophagy.

[36] Bellomo F., Signorile A., Tamma G., Ranieri M., Emma F., De Rasmo D. (2018). Impact of atypical mitochondrial cyclic-AMP level in nephropathic cystinosis. Cell. Mol. Life Sci..

[37] De Rasmo D., Signorile A., De Leo E., Polishchuk E.V., Ferretta A., Raso R., Russo S., Polishchuk R., Emma F., Bellomo F. (2019). Mitochondrial Dynamics of Proximal Tubular Epithelial Cells in Nephropathic Cystinosis. Int. J. Mol. Sci..

[38] Kalatzis V., Serratrice N., Hippert C., Payet O., Arndt C., Cazevieille C., Maurice T., Hamel C., Malecaze F., Antignac C. (2007). The ocular anomalies in a cystinosis animal model mimic disease pathogenesis. Pediatr. Res..

[39] Romero-Ferrero F., Bergomi M.G., Hinz R.C., Heras F.J.H., de Polavieja G.G. (2019). idtracker.ai: Tracking all individuals in small or large collectives of unmarked animals. Nat. Methods.

