# Supplementary material for: Renal and Extra Renal Manifestations in Adult Zebrafish Model of Cystinosis
Source: Int J Mol Sci. 2021 Aug 30;22(17):9398. doi: 10.3390/ijms22179398 (PMC8430996; doi:10.3390/ijms22179398)
Supplement: Supplementary file 1 [file ijms-22-09398-s001.zip › ijms-1340632-supplementary.pdf]

Supplementary Data

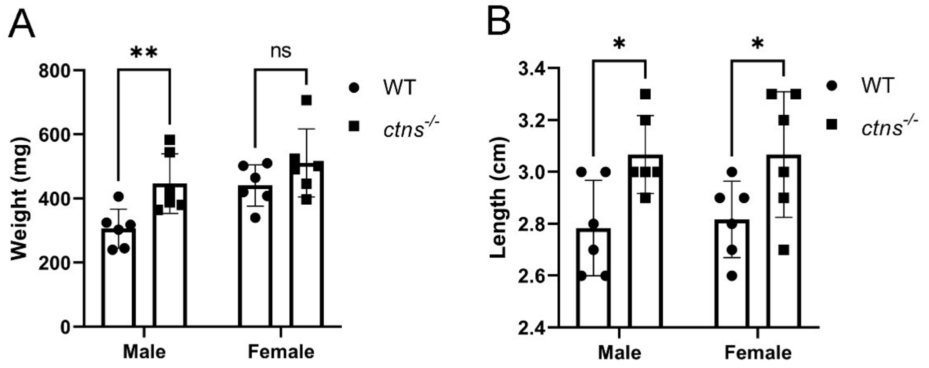

Supplementary Figure S1. Body weight and length measurement in wild-type and *ctns*$^{-/-}$ zebrafish. (A-B) *ctns*$^{-/-}$ male zebrafish present increased (A) body weight when compared with wild-type, while (B) body length is increased in *ctns*$^{-/-}$ zebrafish compared with wild-type in both genders. Each dot represents one zebrafish, for a total of n=6 wild-type and n=6 *ctns*$^{-/-}$ of 18-month-old zebrafish. Two-way ANOVA with Fisher's least significant difference (PLSD) test: *p <0.05; **p <0.01; ns: not significant.
